# PLAGL1 overexpression induces cytoplasmic DNA accumulation that triggers cGAS/STING activation

**DOI:** 10.1111/jcmm.70130

**Published:** 2024-10-04

**Authors:** Cheng Li, Lingyan Qiao, Juan Ge, Sicui Hu, Hongxiu Yang, Conghui Hu, Tang Li

**Affiliations:** ^1^ Department of Pediatric Endocrinologic and Genetic and Metabolic Diseases Qingdao Women and Children's Hospital Qingdao China

**Keywords:** cGAS/STING, DNA senser, PLAGL1, type 1 diabetes mellitus

## Abstract

Pancreatic β‐cell damage mediated by apoptosis is believed to be a main trigger of type 1 diabetes mellitus (T1DM), which is proposed as an organ‐specific autoimmune disease mediated by T cells. Nonetheless, the fundamental origins of T1DM remain uncertain. Here, we illustrate that an increase in PLAGL1 expression induces β‐cell apoptosis, as evidenced by mitochondrial membrane impairment and nucleolar degradation. The gene expression levels from cDNA samples were determined using qRT‐PCR method. Western blot and Co‐immunoprecipitation were applied for protein expression and interactions, respectively. Flow cytometry and TUNEL assay were used to detect pancreatic β cell apoptosis. Female NOD/LtJ mice with recent‐onset T1DM has been used in *in vivo* studies. Glucose‐stimulated insulin secretion (GSIS) and glucose tolerance test (GTT) method is used for islet function assessment. Haematoxylin and Eosin (H&E) and Immunohistochemistry (IHC) were performed to evalute histological improvement of islet beta. Subsequent cytoplasmic DNA accumulation triggers DNA senser, the cyclic guanosine monophosphate‐AMP synthase (cGAS)‐stimulator of interferon genes (STING) pathway. STING activation further stimulates downstream IRF3 and NF‐kB pathways, thus boost type‐I interferon signalling and NF‐kB mediated inflammation. These findings elucidate a molecular mechanism linking PLAGL1 induced cell apoptosis to type‐I interferon signalling and suggest a potential benefit for targeting cGAS/STING in T1DM treatment.

## 1 INTRODUCTION

Type 1 diabetes mellitus (T1DM) has been proposed as an organ‐specific autoimmune disease mediated by T cells. It is characterized by the gradual destruction of pancreatic beta‐cells, resulting in a complete deficiency of insulin and hyperglycemia.^1^ Additionally, recent studies suggest that inflammatory mediators mediated by innate immunity play a significantly broader role in T1DM than previously believed.^2^ Inflammation contributes to both the early and late stages of disease progression, including the initiation and amplification of adaptive immune responses against pancreatic beta cells, as well as the stabilization and maintenance of the inflammatory environment.^2^

Ultimately, these inflammatory mediators contribute to the suppression of beta‐cell function and subsequent apoptosis, as well as peripheral insulin resistance.^3^, ^4^ The primary causes leading to inflammation and the death of pancreatic islet cells in T1DM are not fully established. It is believed to result from autoimmune destruction of pancreatic islet cells, with individuals susceptible to genetic factors. Environmental triggers, such as viruses, may also play a role in the development of T1DM.^5^ Insulin enzyme replacement therapy (ERT) remains the mainstay therapeutic approach; however, it is not adequate for promoting islet regeneration or reversing autoimmune damage associated with T1DM.

As a sensor of cytosolic DNA, cyclic GMP‐AMP synthase (cGAS) initiates innate immune responses by generating the second messenger cyclic GMP‐AMP (cGAMP), which then binds to and activates the stimulator of interferon genes (STING). Upon activation, STING recruits TANK binding kinase 1 (TBK1), leading to the phosphorylation of interferon regulatory factor 3 (IRF3) and subsequent induction of type I interferon (IFN) and chemokines such as CCL5 and CXCL10.^6^, ^7^, ^8^, ^9^ The activated cGAS‐STING pathway plays a crucial role in anti‐tumour immunity by facilitating T cell priming.^10^, ^11^ However, the involvement of the STING pathway in the development of autoimmune diseases remains incompletely understood.

PLAGL1, also recognized as Zac1, functions as a zinc finger transcription factor involved in inhibiting cell proliferation by regulating both cell cycle arrest and apoptosis.^12^, ^13^, ^14^ Positioned on chromosome 6q24, PLAGL1 is an imprinted gene predominantly expressed paternally. Alterations in this gene locus, such as paternal duplication or loss of methylation at the PLAGL1 DMR, precipitate transient neonatal diabetes mellitus (TNDM) due to heightened PLAGL1 expression.^15^, ^16^, ^17^, ^18^, ^19^, ^20^ This overexpression of PLAGL1 seems to diminish beta cell mass in neonates by exerting apoptotic and/or anti‐proliferative effects.^21^ This phenomenon likely stems from PLAGL1's ability to modulate the expression of PPARG and PACAP1‐R, pivotal regulators of beta cell proliferation and insulin secretion, respectively.^22^ Additionally, elevated glucose levels can induce PLAGL1 expression in rodent beta cell lines as well as primary mouse islets.^23^ Notably, overexpression of PLAGL1 in rodent beta cell lines impedes insulin secretion,^24^ while in mice, it mirrors the early‐onset diabetes observed in TNDM patients.^25^ Furthermore, induced PLAGL1 expression prompts a reduction in glucose‐stimulated proinsulin biosynthesis despite an elevation in insulin mRNA levels^23^ suggesting a potential negative regulatory role of PLAGL1 on the translational machinery and ultimately on the efficiency of insulin mRNA translation.

The PLAGL1 gene encoded zinc finger protein Zac1 serves as both a transcription factor and a cofactor for other regulatory proteins.^21^ Being an imprinted gene, PLAGL1 is widely expressed across human organs from embryo to adult stages, and its aberrant expression has been linked to various human tumours.^26^ Previous reports^27^ indicate that the most common cause of TNDM is the overexpression of the PLAGL1 gene in the imprinted locus on chromosome 6q24. Infants with 6q24‐TNDM typically develop insulin‐dependent diabetes shortly after birth, followed by gradual improvement and eventual remission of the condition by 18 months of age. Studies suggest^24^ that PLAGL1 (encoded by the PLAGL1 gene) diminishes insulin secretion in mice by reducing Rasgrf1 expression. In TNDM patients, the function of pancreatic islet β cells is temporarily suppressed without significant damage, partially explaining the eventual alleviation of symptoms. In contrast, pancreatic β‐cell apoptosis is considered the final and most critical stage in the progression of T1DM, resulting from the irreversible elimination of β cells mediated by autoreactive T cells.^28^

PLAGL1 is hypothesized to act as a tumour suppressor by triggering cell cycle arrest and apoptosis through its interactions with partner proteins. It binds with p53, forming a complex that enhances the transcription of p21, resulting in the production of the corresponding protein. This protein, in turn, halts the cell cycle's progression.^21^ However, it remains uncertain whether the PLAGL1/p21 signalling pathway contributes to islet β‐cell damage and apoptosis in T1DM patients, along with its associated mechanism. Our prior research revealed that heightened PLAGL1 expression in pancreatic β‐cells leads to elevated levels of p21 and p53, correlating with reduced cell proliferation and increased apoptosis. Moreover, we observed the presence of GADA (glutamic acid decarboxylase autoantibodies, one of the anti‐islet autoantibodies resulting from β‐cell destruction mediated by T‐cells) in T1DM patients with PLAGL1 overexpression, exhibiting clinical features such as heightened insulin dependence, ineffectiveness of sulfonylureas, and pancreatic β‐cell apoptosis.^12^, ^13^, ^14^ Consequently, this study aims to delve deeper into the effects of PLAGL1 overexpression on pancreatic β‐cell function and elucidate the underlying mechanisms.

## 2 MATERIALS AND METHODS

### 2.1 Transfection and titre identification of lentiviral vector

Lipofectamine 2000 was used to transfect HEK293T cells with lentiviral vectors with low expression of PLAGL1 gene, and then the transfection efficiency was monitored under a fluoroscope after 24–72 h. The cells were then collected and the viral titre was calculated by TCID50 method. Two transfection systems were used to transfect HEK293T cells with high expression vectors: a. pLVX‐IRES‐ZsGreen, pMDLgPRE, pRSV‐Rev, pCMV‐VSV‐G; b. pLVX‐PLAGL1‐IRES‐ZsGreen, pMDLgPRE, pRSV‐rev, pCMV‐VSV‐G. After 72 h of transfection, supernatant was collected by centrifugation to remove cells and cell debris. Lentivirus was concentrated by using polyethylene glycol (PEG) precipitation and the supernatant was discarded, then the viral titre was calculated by TCID50 method after resuspension.

### 2.2 Cell

The pancreatic β‐cell line NIT‐1 was purchased from ATCC (NO. CRL‐2055) and cultured in complete Dulbecco Minimum Essential Medium (DMEM) supplemented with 25 mM glucose, 15 mM HEPES, 1 mM sodium pyruvate, 2 mM L‐glutamine, 2 g/L sodium bicarbonate, 100 mg/L Penicillin/Streptomycin, 10% heat‐inactivated foetal calf serum (FCS) which were adjusted to pH 7.2, and maintained in 75 cm^2^ tissue culture flasks at 37°C in a humidified atmosphere of 5% CO_2_ incubators. Cells were allowed to attach to the flask. Cell culture medium was exchanged every 48 h and cells were passaged at weekly intervals by trypsination. Harvesting and passaging of the NIT‐1 cells were accomplished by detaching, aspirating and separating the adherent cells by mechanical agitation, followed by incubation with 0.25% trypsin and 0.02% EDTA in D‐Hank's solution (pH 7.2) for 1–2 min. PLAG1 and Vector plasmids were transduced into NIT‐1 cells and incubated for 6 days. The survival rate of NIT‐1 cells transduced with Vector plasmid on day 4 was normalized and analysed by CCK‐8 assay.

### 2.3 Enzyme linked immunosorbent assay (ELISA) for insulin level

The concentration of insulin protein in the supernatant of transfected NIT‐1 islet β cell line was quantified by using commercial Mouse Insulin ELISA Kit (Beyotime, PI602, Ultrasensitive) according to the manufacture' instruction.

### 2.4 qRT‐PCR for genes expression

The supernatant of transfected cells was discarded, and 1 mL Trizol (Invitrogen, 15596026CN) was added into the cell pellet. Total RNA was extracted and cDNA was synthesized from 500 ng of total RNA using Revert Aid First Strand cDNA Synthesis Kit (Thermo Scientific, K1621), The gene expression levels from cDNA samples were determined using SYBR Select Master Mix (Thermo Scientific, Applied Biosystems, 4472918). Primer sequences for quantitative PCR assays are listed in Table S1. All assays were conducted in triplicate and normalized to GAPDH expression.

### 2.5 Flow cytometry for pancreatic β cell apoptosis

Cells were dissociated by 0.25% trypsin and washed twice with PBS. After adding 100 μL binding buffer containing Annexin‐V‐FITC (20 μg/mL at concentration), the cells were incubated at room temperature in the dark for 20 min, followed by adding 5 μL propidium iodide (PI) (50 μg/mL at concentration) for another 5 min incubation in the dark. Flow cytometry was performed immediately after adding 400 μL binding buffer, to detect the apoptosis of pancreatic islet β cells.

### 2.6 TUNEL assay for pancreatic β cell apoptosis

Cellular DNA fragmentation is a hallmark of apoptosis, thus TUNEL assay was used to detect in situ DNA strand breaks. The cells were seeded onto 24‐well plate and rinse with PBS solution for 5 min, then the cells were fixed with pre‐cooled 4% paraformaldehyde at 4°C for 30–60 min, followed by washing three times in PBS, 5 min each wash. Removed the solution and incubated the cells in PBS solution containing 0.1% Triton X‐100 for 2–5 min in ice bath. Afterwards, covered the cells with 50 μL of 3% H_2_O_2_ and incubated at RT for 10 min in the dark. Removed the solution and rinsed with PBS, proceeded by applying 50 μL TUNEL reaction mix into the well and incubated at 37°C for 60–90 min in the dark. In the end, rinsed the cells with PBS and observed the cell counts under a fluorescence microscope.

### 2.7 Western blot and Co‐immunoprecipitation for protein expression and interaction

The supernatant of transfected cells was discarded, RIPA lysis buffer was used. And protein concentration was measured using BCA assay according to the protocol (Thermo Scientific, Pierce™ BCA Protein Assay Kits, Cat. 23225). For Co‐immunoprecipitation, the cells were lysis for 30 min on ice, and the cell lysate was collected, followed by adding specific antibodies and incubating overnight. Ten microlitre of pretreated protein A agarose beads were added to above cell lysate and incubated for 2–4 h at 4°C with gentle shaking. After the immunoprecipitation reaction, the agarose beads were pelleted by centrifugation, and then the supernatant was discarded. Washed the agarose beads with 1 mL lysis buffer, and boiled the beads for 5 min after adding loading buffer. Western blot or mass spectrometry was performed to analyse protein–protein interactions. Primary antibodies used in western blot tests include PLAGL1(Abcam, ab129063), STING (Abcam, ab239074), IRF (Abcam, ab271043) and phosphorylated IRF (Abcam, ab309088), p65 (Abcam, ab32536) and phosphorylated p65 (Abcam, ab76302).

### 2.8 Lentivirus transfection of NOD mice

The 12‐16‐week‐old female NOD/LtJ mice with recent‐onset T1DM were randomly divided into four groups: (1) Normal control group: tail vein injection with 8.4% bicarbonate solution; (2) Empty vector control group: the tail vein was injected with empty vector containing 5 × 10^8^ pLVX‐IRES‐ZsGreen1 particles; (3) PLAGL1 overexpression group: tail vein injection with virus solution containing 5 × 10^8^ pLVX‐PLAGL1‐IRES‐ZsGreen1 particles; (4) PLAGL1 inhibition group: tail vein injection with virus solution containing 5 × 10^8^ pSUPER‐EGFP‐P1 particles. The mice were reared until 25–26 weeks after injection.

### 2.9 Islet function assessment in mice

GSIS (glucose‐stimulated insulin secretion) method was used to investigate the insulin secretion function of NOD mice. Pancreatic β‐cells recognize extracellular glucose concentration and secrete insulin as required at a given time. Briefly, NOD mice were fasted for 8 h after transfection, and glucose solution was injected intraperitoneally at 2 g/kg. Blood was collected from medial canthus at pre‐injection, 15, 30 and 60 min after injection, and insulin level was measured by ELISA as previously mentioned.

The GTT (glucose tolerance test) method was performed to evaluate the function of pancreatic islets in NOD mice. In order to assess glucose homeostasis and determine the beta cell insulin secretory capacity in mice *in vivo*, intraperitoneal GTT was used in this study for measuring blood glucose at given time. As mentioned above in GSIS, NOD mice were fasted for 8 h after transfection, and glucose solution was injected intraperitoneally at 2 g/kg. Blood was collected at pre‐injection, 15, 30, 60 and 120 min after injection. Haematoxylin and Eosin (H&E) staining was used to observe the islet morphology of mice. The pancreas of transfected NOD mice were fixed in 4% paraformaldehyde for 24 h, and then the paraffin‐embedded pancreas sectioning for HE staining to evaluate the islet morphology.

### 2.10 Immunohistochemistry

Immunohistochemistry (IHC) was performed to determine the number of islet β cells in mice. Paraffin‐embedded pancreas section was stained by IHC, followed by scanning with the digital pathological microtome and no less than four fields of view were observed for each slide. Finally, the proportion of positive insulin staining cells was counted with Image‐Pro Plus 6.0 software, which was the positive rate of pancreatic β cells.

### 2.11 Statistical analysis

We did not use specific sample size calculation methods. Any technically validated data were not excluded. Data were analysed statistically using GraphPad Prism software (Ver. 6; La Jolla, CA, USA) using the methods indicated in the legend of each figure. Two‐sided Student's *t*‐test and one‐way ANOVA followed by Tukey–Kramer post‐hoc test were used to compare the data among two and more than two groups, respectively. Log‐rank test was used to evaluate the survival curve data. A *p* < 0.05 was considered statistically significant.

## 3 RESULTS

### 3.1 PLAGL1 overexpression induce pancreatic β cell apoptosis revealed by mitochondrial damage

**FIGURE 1:**
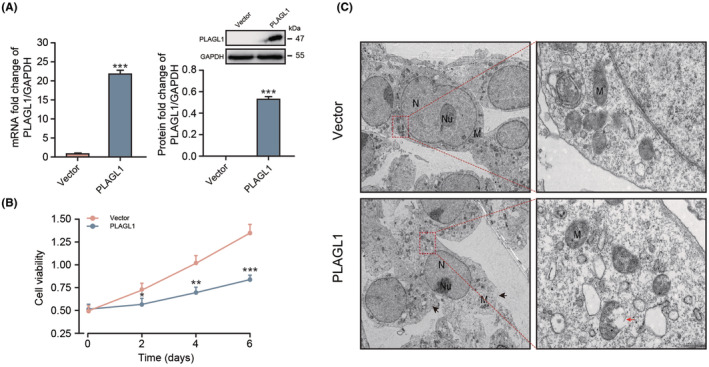
PLAGL1 overexpression induce pancreatic β cell apoptosis. (A) PLAGL1 mRNA transcription and PLAGL1 protein translation in β cell upon PLAGL1 overexpression plasmid transfection, (B) β cell viability upon transfection of PLAGL1 or empty vector, (C) sup‐cellular structure of PLAGL1 or empty vector transfected β cells (N: Nucleus, Nu: Nucleolus, M: Mitochondrial). *, **, ***p < 0.05, 0.01, 0.001, respectively.

Previous studies reported that The PLAGL1 gene encodes a homonymous zinc finger protein that promotes cell cycle arrest and apoptosis in multiple cell types. However, it is still unclear how PLAGL1 works in β cells. To evaluate the effects of PLAGL1 on pancreatic β cells, we have ectopically expressed murine PLAGL1 in murine NIT‐1 β cells (Figure 1A). Transduced NIT‐1 β cells were incubated over 6 days, and viable cell counting has been performed using CCK‐8 cell viability assay. The living cell number was significantly reduced overtime in PLAGL1 transduced group comparing to the mock treatment group (Figure 1B), which is in line with previous studies, indicating detrimental effect of PLAGL1 overexpression is not cell type dependent.

The detrimental effect of PLAGL1 overexpression on cell maintenance in culture could be due to at least two mechanisms, cell cycle arrest or cell death. To investigate the effect of PLAGL1 in cell death, we have further detailed subcellular structure perturbation of NIT‐1 β cell upon PLAGL1 overexpression, by using electron microscopy. PLAGL1 overexpression induce NIT‐1 cell mitochondrial membrane damage (black arrow), and the nucleolus (Nu) was not obvious as the typical spherical body, while control vector transduced NIT‐1 β cells were normal (Figure 1C). The results suggested that PLAGL1 overexpression in NIT‐1 β cells induces cell apoptosis revealed by mitochondrial membrane damage (black arrow), and the nucleolus morphologic change.

### 3.2 PLAGL1 mediated intracellular membrane damage leads to cytoplasmic DNA accumulation

**FIGURE 2:**
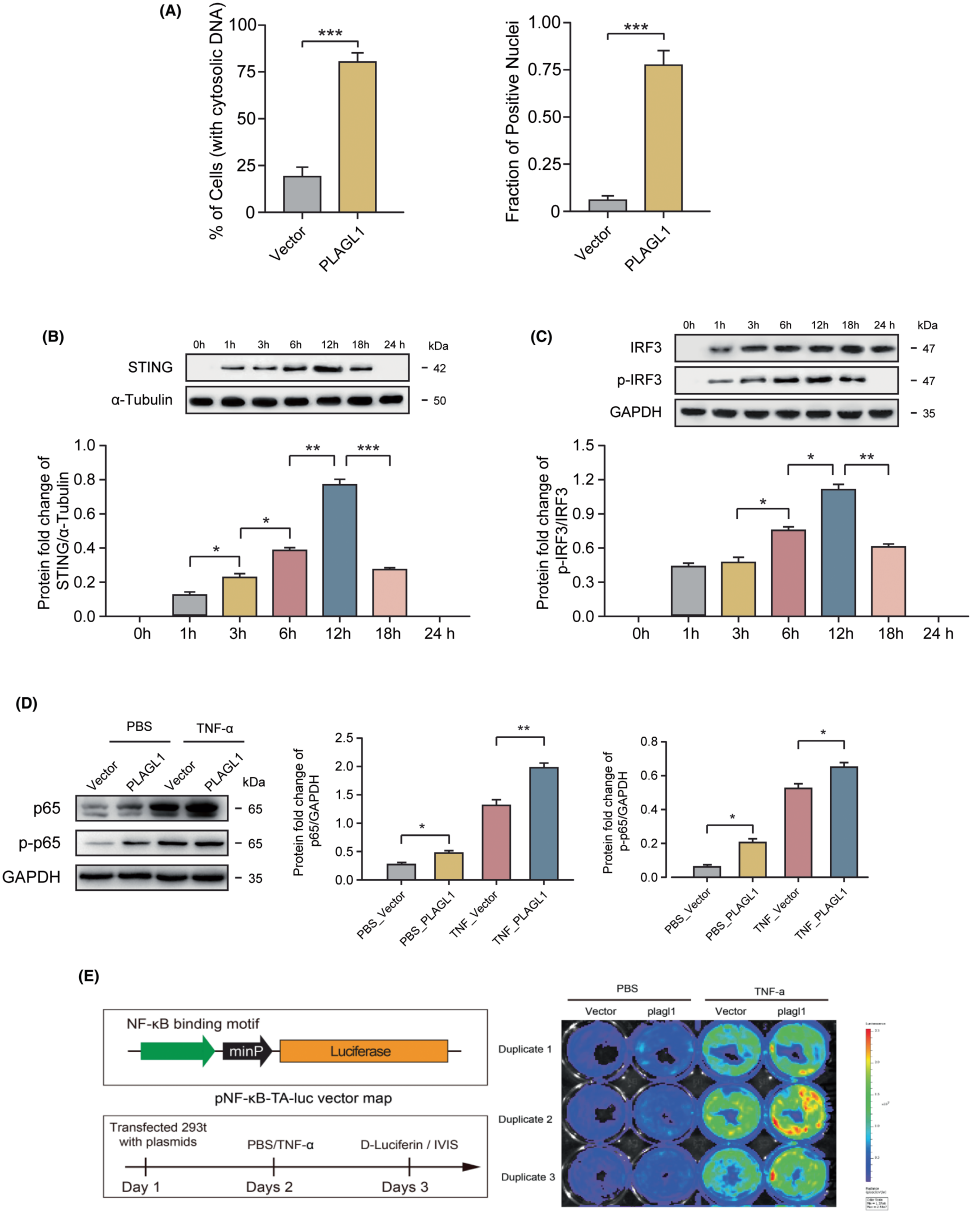
PLAGL1 overexpression Induces Cytosolic DNA Accumulation and triggers cGAS/STING pathway activation. (A) Cytosolic DNA positive NIT‐1 β cells and the fractions of positive nuclei upon PLAGL1 plasmid or vector transfection, (B) STING expression level measured by western blot upon treatment, (C) IRF and phosphorylated IRF level measured by western blot upon treatment, (D) p65 and phosphorylated p65 level measured by western blot upon treatment, (E) schematic structure of NF‐kB reporter and experiment design (left), and bioluminescence in differently treated cells. *, **, ***p < 0.05, 0.01, 0.001, respectively.

As shown above, murine PLAGL1 overexpression mediates mitochondrial damage and the nucleolus perturbation. We reasoned that the subcellular structure damage would result the accumulation of fragments of damaged genomic DNA that ended up moving to the cytoplasm, as well as mitochondrial DNA release. We performed Hoechst staining and imaging upon PLAGL1 overexpression over 48 h. PLAGL1 transduced NIT‐1 β cells displayed discrete, microsomal appearing cytoplasmic granules of Sir‐Hoechst labelled DNA in the periphery of the nucleus (Figure 2A), indicating significant cytosolic DNA accumulation was induced by PLAGL1 expression.

### 3.3 PLAGL1 overexpression mediated cytoplasmic dsDNA accumulation triggers cGAS/STING pathway activation

Upon recognition of cytosolic DNA, cGAS catalyses the formation of cyclic guanosine monophosphate (GMP)‐adenosine monophosphate (AMP) (cGAMP), which subsequently activates the adaptor protein stimulator of interferon genes (STING) and further induces phosphorylation and nuclear translocation of IFN transcriptional regulatory factors TANK‐binding kinase 1 (TBK1) and IFN regulatory factor 3 (IRF3). Phosphorylated IRF3 homodimerizes and then is translocated into the nucleus, where it increases the expression of type I IFNs, thereby leading to antiviral immune responses. STING also upregulates inflammatory cytokines and chemokines by activating the kinase IKK, which phosphorylates and inactivates the IkB family of inhibitors of the transcription factor NF‐kB.

To further investigate the involvement of PLAGL1 in STING pathway mediated by cytosolic DNA accumulation, we first transduced NIT‐1 β cells with PLAGL1 overexpression vector and we then evaluated the status of STING in NIT‐1 β cells. After PLAGL1 transduction, NIT‐1 β cells were collected and the STING activation were monitored by immunoblotting. Upon PLAGL1 overexpression, the level of STING increased significantly (Figure 2B). As mentioned above, STING activation subsequently leads to phosphorylation of IRF3 that in turn leads to type I interferon activation, and at the same time STING activation also mediates NF‐KB activation. To further confirm the downstream signalling of STING activation, immunoblotting was carried out to quantify phosphorylation of IRF3 and NFκB subunit p65 (RelA) expression and p65 phosphorylation(p‐p65) upon PLAGL1 expression in NIT‐1 β cells. As shown, the phosphorylation of IRF3 triggered significantly upon PLAGL1 overexpression (Figure 2C). And as expected, p65 phosphorylation(p‐p65) was also increased by ectopic PLAGL1 expression with or without TNF stimulation (Figure 2D). p65 phosphorylation and dimerization could initiate NFκB translocation and further activate its target genes.

To further validate NF‐κB activation mediated by PLAGL1, we transfected NF‐κB luciferase reporter plasmid (pNF‐KB‐TA‐Luc) into PLAGL1 overexpressed cells, and the luciferase assay clearly showed that PLAGL1 overexpression induced activation of NF‐κB (Figure 2E), which is in line with immunoblotting observation, further confirmed that PLAGL1 can activate NFκB and its down‐stream target genes.

### 3.4 Type I interferon gene module is triggered upon PLAGL1 overexpression

**FIGURE 3:**
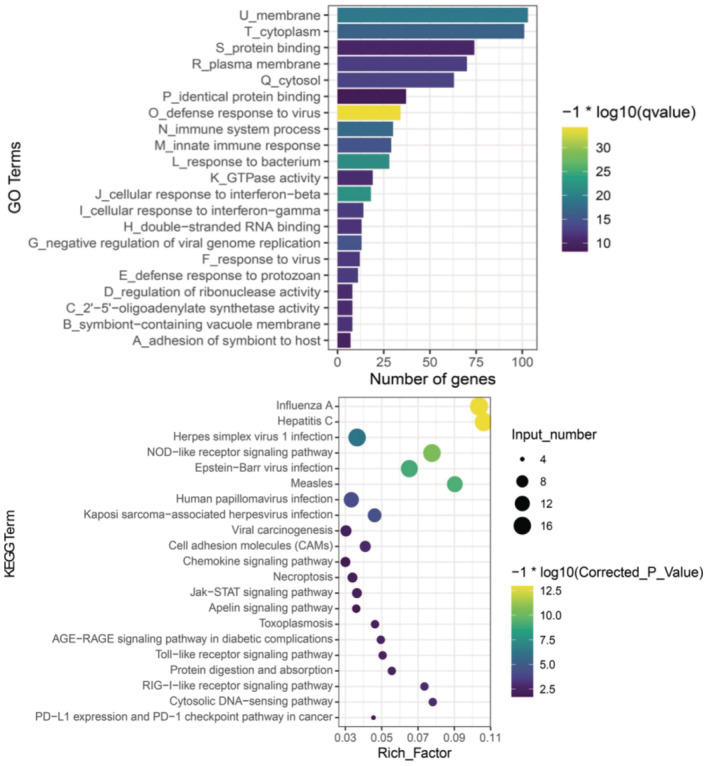
GO term enrichment and KEGG pathway analysis for the identification of key genes and pathways that regulated by PLAGL1 knock down.

Both IRF3 and NF‐kB are master transcription factors that involved in large amount of target gene expression regulation and varies cellular processes. To mapping the systemic regulatory network of PLAGL1 in β cells, we created PLAGL1 knockdown NIT‐1 β cells and PLAGL1 overexpression NIT‐1 β cells, and total mRNA were purified followed by library preparation. And cDNA libraries were then sequenced to measure gene expression levels. We then applied GO term enrichment and KEGG pathway analysis for the identification of key genes and pathways (Figure 3). The functional enrichment analysis showed PLAGL1 overexpression induced differential expression genes enriched in the inflammatory response similar to a viral infection.

**FIGURE 4:**
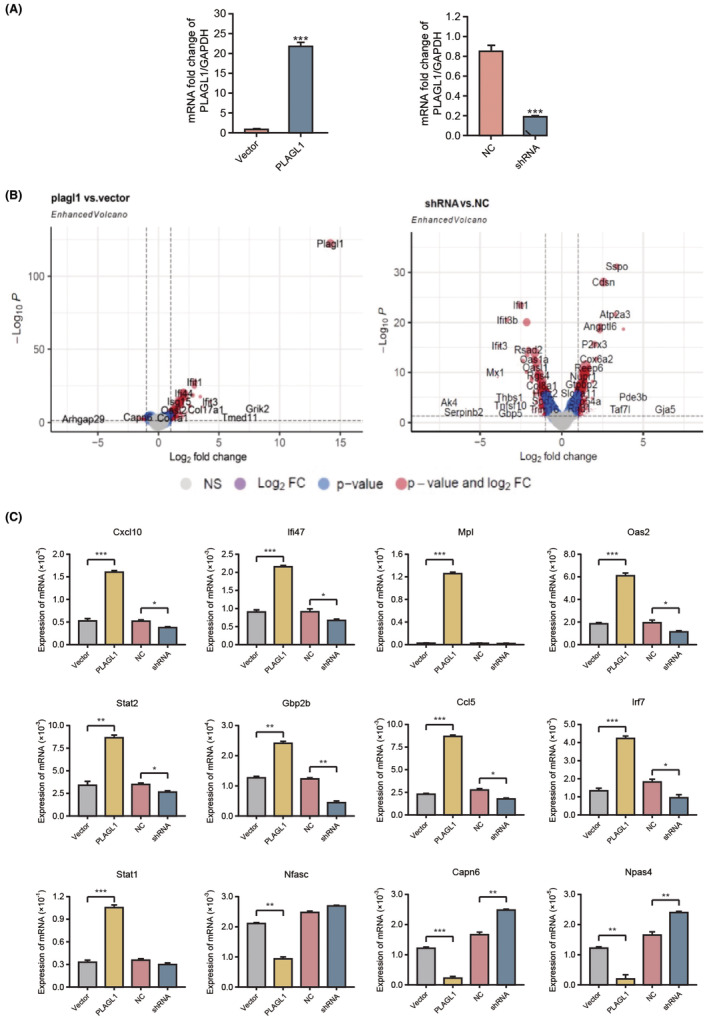
cGAS/STING pathway activation upregulates type I interferon gene module. (A) PLAGL1 overexpression or knock‐down as revealed by quantitative PCR, (B) Volcano plot to show PLAGL1 regulated genes upon PLAGL1 overexpression or knock‐down, (C) qPCR to quantify the selected IFN response genes. *, **, ***p < 0.05, 0.01, 0.001, respectively.

To figure out the individual genes that contributed to the pathway activation, NIT‐1 β cells were transfected with PLAGL1 overexpression plasmid or siRNA to over express or knock down PLAGL1 (Figure 4A), and followed by mRNA sequencing. Volcano plot was generated, and as expected IFN stimulated gene module were upregulated upon PLAGL1 overexpression, and IFN stimulated genes were down modulated when knock PLAGL1 down by using siRNA (Figure 4B). IFN stimulated gene module is likely downstream of STING activation mediated by cytoplasmic DNA accumulation. The IFN gene module was down‐regulated when PLAGL1 were silenced up shRNA transduction, further confirmed the IFN stimulating function of PLAGL1 in β cells.

To further validate the RNAseq readout, we then perform qPCR to quantify the selected IFN response genes, including Cxcl10, Ifi47, Mpl, Oas2, Stat2, Gbp2b, Ccl5 and Irf7, and the regulation trend of those gene in highly in line with RNAseq data (Figure 4C). These result indicated that PLAGL1 in β cells mediates strong IFN response gene expression, which potentially leads inflammation and induce islet damage.

### 3.5 PLAGL1 overexpression impair islet function in vivo

**FIGURE 5:**
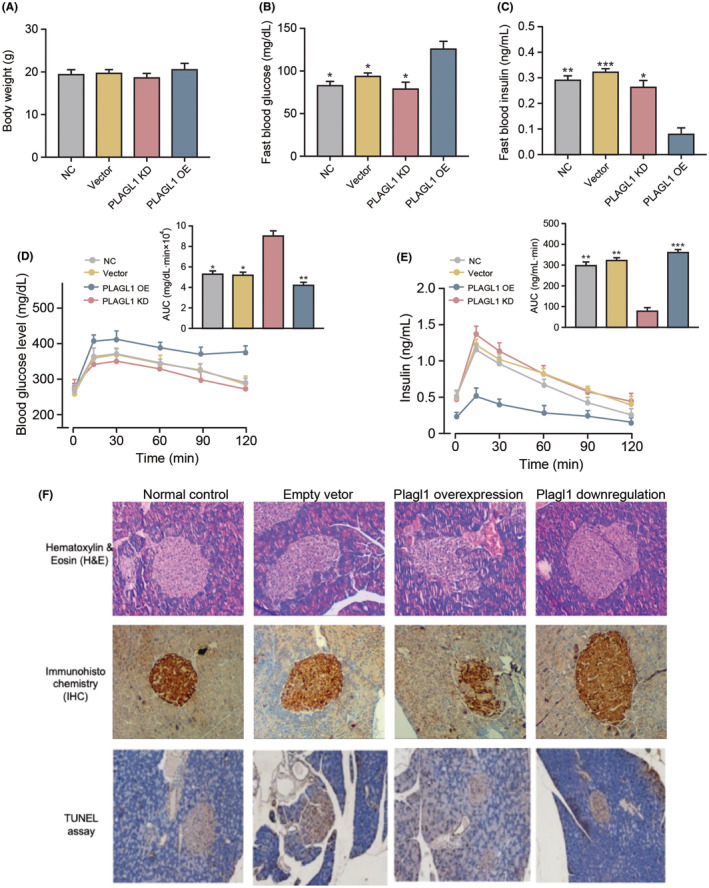
PLAGL1 overexpression impair islet function in vivo. (A). PLAGL1 expression in normal, PLAGL1 KD and PLAGL1 OE mice; (B) body weight of different treated groups of mice; (C) fast blood glucose measures of different treated groups of mice; (D) fast blood insulin level measures of different treated groups of mice; (E) dynamic curve of blood glucose level in different treated groups of mice; (F) dynamic curve of insulin level in different treated groups of mice; (G). histologic analysis of islet morphology. *, **, ***p < 0.05, 0.01, 0.001, respectively.

Although PLAGL1 overexpression in β cells induces cell apoptosis and mediates inflammation, however, it is not clear how these *in vitro* observations translated in mice model. To study the impact of PLAGL1 *in vivo*, we generated mouse model with PLAGL1 overexpression or PLAGL1 knockdown in β cells. And we then applied series of assessments to verify PLAGL1 impact on islet function *in vivo*. The PLAGL1 expression in normal, PLAGL1 KD and PLAGL1 OE mice were showed in Figure 5A. From the biochemical examination, PLAGL1 overexpression mice group showed similar body weight as other groups (Figure 5B), significant higher fast blood glucose (Figure 5C) and lower fast blood insulin level (Figure 5C), indicated hyperglycemia. Then we further evaluate if PLAGL1 overexpression induce impaired glucose tolerance in mice. In addition to PLAGL1 overexpressed group, other three groups (including normal control, empty vector and PLAGL1 down‐regulation) displayed a typical GTT dynamic curve with normal glucose tolerance (Figure 5D), in which, a rapid in blood glucose reaching its peak 15 min after the glucose challenge, followed by subsequent glucose uptake leads to a gradual decrease of the blood glucose concentration to the normal level after approximately 90 ~ 120 min. GTT dynamic curve together with the elevated cumulative AUC in Plagl1 overexpressed group(Figure 5E) can pinpoint the impaired glucose tolerance. Moreover, a loss of the glucose‐induced insulin release corresponds to initial spike response further supporting abnormal glucose homeostasis and impaired islet functions in PLAGL1 overexpressed mice (Figure 5F).

We performed histologic analysis for islet function by measuring islet morphology, islet β cell number, and β cell apoptosis in pancreas (Figure 5G). An irregular morphology or destruction of islet, fewer cells staining positive for insulin and more cell apoptosis can be seen in PLAGL1 overexpressed mice, as compared with untreated control mice or PLAGL1 down‐regulated mice.

## 4 DISCUSSION

PLAGL1 is a transcription factor, which transcriptionally regulates its target genes, including p53, p21, PPARγ and PACAP1‐R, by binding to differential DNA motifs.^22^, ^29^, ^30^ Its role as an activator or suppressor hinges on the specific binding patterns it adopts. For instance, while PLAGL1 typically binds to G4C4 palindromic DNA elements as a monomer, facilitating transactivation, similar binding to repeat elements transforms the protein into a repressor. PLAGL1 exerts its influence on cell cycle arrest through multiple pathways. On one front, it engages with p53, binding either to its N‐terminal domain or weakly to its C‐terminal fragment. The coactivator function of PLAGL1 in various cell models is contingent upon the presence of p53 as a co‐factor.

Both p53 and PLAGL1 trigger the expression of PACAP1‐R, an upstream receptor that stimulates AP‐1, through their engagement with ligands. In specific cell contexts, AP‐1 activation further prompts programmed cell death. Additionally, both PLAGL1 and p53 induce p21, which inhibits the transcription of cyclin A, culminating in G1 arrest. Notably, PLAGL1 also regulates the p21 promoter via a p53‐independent pathway. It binds to amino acids 481–683 of Sp1, a transcription factor involved in modulating p21 transcription. Furthermore, PLAGL1 upregulates PPARγ, which halts cell cycle progression by inducing p21 expression.^14^, ^31^ Consequently, PPARγ stimulation triggers apoptosis through both the caspase‐dependent and extrinsic pathways.

More recently, the Tcf4 gene has been identified as another direct target of PLAGL1. Experiments using a mouse neurogenesis cell model have shown that PLAGL1 overexpression leads to transactivation at the Tcf4 gene locus, resulting in p57‐mediated G1 cell cycle arrest.^32^ PLAGL1 has been implicated among the roughly 50 susceptibility genes for T1DM.^33^ Elevated levels of PLAGL1 protein during fetal and neonatal stages contribute to a reduction in pancreatic β‐cell mass through apoptosis or cell cycle arrest, consequently impairing insulin sensitivity and release.^34^

In our current investigation, the precise molecular pathway underlying the initiation of cell apoptosis remains unresolved, despite previous studies indicating that PLAGL1 regulates the transcription of PPARγ, PACAP1‐R and Rasgrf1, genes closely associated with carbohydrate metabolism.^22^, ^24^ Specifically, PLAGL1 promotes the expression of PPARγ and PACAP1‐R in pancreatic islets, which respectively act as inhibitors of β‐cell proliferation and insulin secretion.^19^, ^22^

Interestingly, T‐cells isolated from non‐obese mice affected by this disease displayed higher growth/survival ratios but exhibited lower levels of PLAGL1 expression compared to T‐cells from non‐diabetic mice.^33^ This heightened proliferative capacity is likely linked to the autoimmune destruction of pancreatic β‐cells characteristic of T1DM. However, the connection between PLAGL1 overexpression and autoimmunity remains elusive.

In the present study, we observed damage to the membrane structures of mitochondria and the nucleolus upon ectopic overexpression in β‐cells, although the precise mechanism remains unclear. This damage may be attributed to the loss of membrane potential during apoptosis. Collectively, these findings suggest that PLAGL1 expression diminishes mitochondrial membrane potential, possibly via uncoupling of the electron transport chain. Consequently, the disruption of mitochondrial and nucleolar integrity leads to the leakage of genomic DNA and mtDNA. The accumulation of cytoplasmic DNA triggers DNA sensors such as the cGAS/STING pathway. As a pattern recognition receptor, STING is capable of recognizing cyclic dinucleotides in the cytoplasm, subsequently recruiting TANK‐binding kinase 1 (TBK1) and interferon regulatory factor 3 (IRF3), promoting the expression of type‐I interferons, and activating innate immune responses. At the same time, the activation of STING may also promote the activation of the NF‐κB signalling pathway through other mechanisms, further enhancing the inflammatory response. Given the potential role of the cGAS/STING pathway in autoimmune diseases, targeted therapy against this pathway may provide new strategies for the treatment of autoimmune diseases such as T1DM. By inhibiting excessive immune responses, promoting immune tolerance and combining with other immunoregulatory therapies, it is expected to achieve more effective disease control and treatment.^35^, ^36^ Indeed, our study revealed robust activation of the STING pathway, as evidenced by IRF3 and NF‐kB phosphorylation. Intriguingly, previous research has implicated the cGAS‐STING pathway in diabetic angiopathy, which is associated with lipotoxicity‐induced mitochondrial dysfunction. Thus, our findings align closely with prior discoveries. Moreover, there is evidence indicating that the cGAS‐STING pathway is overactivated in diabetes and its complications.^37^, ^38^, ^39^, ^40^ In brief, the activation of STING plays a pivotal role in type‐I interferon signalling and NF‐κB‐mediated inflammation. As a pattern recognition receptor, STING is capable of recognizing cyclic dinucleotides in the cytoplasm, subsequently recruiting TANK‐binding kinase 1 (TBK1) and interferon regulatory factor 3 (IRF3), promoting the expression of type‐I interferons, and activating innate immune responses. At the same time, the activation of STING may also promote the activation of the NF‐κB signalling pathway through other mechanisms, further enhancing the inflammatory response. Given the potential role of the cGAS/STING pathway in autoimmune diseases, targeted therapy against this pathway may provide new strategies for the treatment of autoimmune diseases such as T1DM. By inhibiting excessive immune responses, promoting immune tolerance and combining with other immunoregulatory therapies, it is expected to achieve more effective disease control and treatment.

While the exact origins of T1DM remain unclear, autoreactive T cells are thought to play a pivotal role, mistakenly targeting and destroying β‐cells. A pressing question arises: what prompts CD8+ T cells to launch an assault on these β‐cells? One significant regulatory pathway implicated is the cGAS/STING pathway, which governs both innate and adaptive immunity, particularly through the type‐I interferon signalling cascade. This cascade is notably heightened in β‐cells following PLAGL1 overexpression.

In summary, our investigation unveils a mechanistic correlation between PLAGL1‐induced apoptosis and inflammation. Elevated PLAGL1 levels induce β‐cell apoptosis, resulting in mitochondrial membrane impairment and nucleolus degradation. Subsequent accumulation of cytoplasmic DNA activates the cyclic guanosine monophosphate‐AMP synthase (cGAS)‐stimulator of interferon genes (STING) pathway. STING activation subsequently enhances downstream IRF3 and NF‐kB pathways, amplifying type‐I interferon signalling and NF‐kB‐mediated inflammation. These findings shed light on the molecular pathway linking PLAGL1‐induced cell death to type‐I interferon signalling and suggest potential therapeutic avenues targeting cGAS/STING in T1DM.

## AUTHOR CONTRIBUTIONS

**Cheng Li:** Conceptualization (equal); data curation (equal); formal analysis (equal); funding acquisition (equal); investigation (equal); methodology (equal); project administration (equal); supervision (equal); writing – original draft (equal); writing – review and editing (equal). **Lingyan Qiao:** Investigation (equal); methodology (equal); writing – original draft (equal); writing – review and editing (equal). **Juan Ge:** Investigation (equal); writing – original draft (equal); writing – review and editing (equal). **Sicui Hu:** Resources (equal); software (equal); writing – original draft (equal); writing – review and editing (equal). **Hongxiu Yang:** Resources (equal); software (equal); writing – original draft (equal); writing – review and editing (equal). **Conghui Hu:** Validation (equal); visualization (equal); writing – original draft (equal); writing – review and editing (equal). **Tang Li:** Validation (equal); visualization (equal); writing – original draft (equal); writing – review and editing (equal).

## FUNDING INFORMATION

This study was funded by Shandong Provincial Natural Science Foundation: Youth Fund (ZR2021QH257).

## CONFLICT OF INTEREST STATEMENT

The authors declare no conflicts of interest.

## Supporting information


**Table S1.** Primers used in this study.

## Data Availability

All data generated are included in the manuscript.
