# Supplementary material for: PLAGL1 overexpression induces cytoplasmic DNA accumulation that triggers cGAS/STING activation
Source: J Cell Mol Med. 2024 Oct 4;28(19):e70130. doi: 10.1111/jcmm.70130 (PMC11451391; doi:10.1111/jcmm.70130)
Supplement: Supplementary file 1 — Table S1. Primers used in this study. [file JCMM-28-e70130-s001.docx]

Supplementary materials

**Table S1** Primers used in this study.

| **Primer name** | **Sequences (5' to 3')** |
| --- | --- |
| Irf7_F | TCCAGTTGATCCGCATAAGGT |
| Irf7_R | CTTCCCTATTTTCCGTGGCTG |
| Oas2_F | TTGAAGAGGAATACATGCGGAAG |
| Oas2_R | GGGTCTGCATTACTGGCACTT |
| Stat2_F | TCCTGCCAATGGACGTTCG |
| Stat2_R | GTCCCACTGGTTCAGTTGGT |
| Cxcl10_F | CCAAGTGCTGCCGTCATTTTC |
| Cxcl10_R | GGCTCGCAGGGATGATTTCAA |
| Mpl_F | GCTTCTCCCAAACATTTGAGGA |
| Mpl_R | AGCAGGTTTCCACACTATCCA |
| Ifi47_F | TCTCCAGAAACCCTCACTGGT |
| Ifi47_R | TCAGCGGATTCATCTGCTTCG |
| Stat1_F | TCACAGTGGTTCGAGCTTCAG |
| Stat1_R | CGAGACATCATAGGCAGCGTG |
| Nfasc_F | CCCCTTGACACTACAGTGCAA |
| Nfasc_R | GCAGCATGACGTTGGAGAAGT |
| Capn6_F | GGACCGAGGTGGTGATTGATG |
| Capn6_R | TCCAGTAGAGCATTCCAAAACTC |
| Npas4_F | CAGATCAACGCCGAGATTCG |
| Npas4_R | GACACCCTTGCGAGTGTAGAT |
| Gapdh_F | AGGTCGGTGTGAACGGATTTG |
| Gapdh _R | GGGGTCGTTGATGGCAACA |
